# Dynamic Changes in Myofibroblasts Affect the Carcinogenesis and Prognosis of Bladder Cancer Associated With Tumor Microenvironment Remodeling

**DOI:** 10.3389/fcell.2022.833578

**Published:** 2022-03-02

**Authors:** YiHeng Du, YiQun Sui, Jin Cao, Xiang Jiang, Yi Wang, Jiang Yu, Bo Wang, XiZhi Wang, BoXin Xue

**Affiliations:** ^1^ Department of Urology, Suzhou Kowloon Hospital, Shanghai Jiaotong University School of Medicine, Suzhou, China; ^2^ Department of Urology, The Second Affiliated Hospital of Soochow University, Suzhou, China; ^3^ Department of Pathology, The Second Affiliated Hospital of Soochow University, Suzhou, China; ^4^ Department of Pathology, Suzhou Kowloon Hospital, Shanghai Jiaotong University School of Medicine, Suzhou, China

**Keywords:** bladder cancer, tumor microenvironment, myofibroblasts, carcinogenesis, therapy responsiveness

## Abstract

Bladder cancer (BLCA) is a tumor that possesses significant heterogeneity, and the tumor microenvironment (TME) plays an important role in the development of BLCA. The TME chiefly consists of tumor cells and tumor-infiltrating immune cells admixed with stromal components. Recent studies have revealed that stromal components, especially cancer-associated fibroblasts (CAFs), affect immune cell infiltration and modulate the extracellular matrix in the TME of BLCA, ultimately impacting the prognosis and therapeutic efficacy of BLCA. Among the subgroups of CAFs, myofibroblasts (myCAFs) were the most abundant and were demonstrated to play an essential role in affecting the prognosis of various tumors, including BLCA. However, the dynamic changes in myCAFs during carcinogenesis and tumor progression have been less discussed previously. With the help of bioinformatics algorithms, we discussed the roles of myCAFs in the carcinogenesis and prognosis of BLCA in this manuscript. Our study highlighted the pathogenesis of BLCA was accompanied by a decrease in the abundance of myCAFs, revealing potential protective properties of myCAFs in the carcinogenesis of BLCA. Meanwhile, the reduced expressions of myCAFs marker genes were highly accurate in predicting tumorigenesis. In contrast, we also demonstrated that myCAFs regulated extracellular matrix remodeling, tumor metabolism, cancer stemness, and oncological mutations, ultimately impacting the treatment responsiveness and prognosis of BLCA patients. Thus, our research revealed the bimodal roles of myCAFs in the development of BLCA, which may be associated with the temporal change of the TME. The in-depth study of myofibroblasts and the TME may provide potential diagnostic biomarkers and therapeutic targets for BLCA.

## Introduction

Bladder cancer (BLCA) is a common cancer of the urinary system with two distinct features, frequent recurrence and heterogeneity in tumor progression (Lokeshwar et al., 2020). Clinically, BLCA can be divided into muscle-invasive bladder cancer (MIBC) and non-muscle-invasive bladder cancer (NMIBC) (Kamoun et al., 2020). Of note, myofibroblasts are present in the vast majority of MIBC sections, suggesting that myofibroblasts play a critical role in the progression and heterogeneity of BLCA. Owing precisely to the highly heterogeneous nature of MIBC, patients with MIBC have limited treatment options and often need to undergo radical cystectomy, which significantly affects patients’ quality of life (Gakis et al., 2017). With the recent development of immunotherapy, immune checkpoint blockade (ICB) therapy is now a guideline-recommended treatment for advanced BLCA when chemotherapy fails (Funt and Rosenberg, 2017). However, ICB therapy still faces many limitations, including multiple adverse effects and unpleasant therapeutic responsiveness (Lopez-Beltran et al., 2021).

The tumor microenvironment (TME) is an intricate system that mainly consists of tumor cells, stromal cells, and tumor infiltrated immune cells (Patnaik et al., 2017). Emerging evidence has shown that stromal components can shape the TME, influence chemotherapy and immunotherapy responsiveness, and promote malignant tumor progression (Yang et al., 2018). Cancer-associated fibroblasts (CAFs) occupy a dominant position in the stromal components of the TME. Recent evidence suggests that CAFs play profound roles in shaping the immune landscape of the TME *via* ECM-regulated immune cell anchorage and trafficking and via suppression of immune activation (Yu et al., 2020). In addition, CAFs-associated remodeling of the TME also plays a crucial role in the chemosensitivity of tumors (Zhang et al., 2020). Thus, the functions of CAFs in the tumor microenvironment are complicated, and further studies on CAFs are critical in the field of cancer research.

With the rapid development of single-cell RNA sequencing (scRNA-seq) techniques, CAFs are now considered to be classified into different subgroups, including inflammatory CAFs (iCAFs), myofibroblasts (myCAFs), and antigen-presenting CAFs (apCAFs) (Elyada et al., 2019). myCAFs have been shown to promote a more aggressive cancer cell phenotype by both *in vivo* and *in vitro* experiments (Otranto et al., 2012). Meanwhile, myCAFs have been demonstrated to greatly impact patients’ prognosis among various types of human cancers (Liu et al., 2016). However, controversy remains, with some studies confirming that animal models of pancreatic cancer after removal of myCAFs exhibit significantly worse prognosis, suggesting that fibroblast reactions could also play a protective role (Özdemir et al., 2014). Therefore, it is essential to investigate the dynamics of myCAFs to study the temporal alterations in the tumor microenvironment.

In the present study, we comprehensively discussed the roles of myCAFs in the carcinogenesis and progression of BLCA with the help of bioinformatics algorithms and immunohistochemical validations. Our research revealed dual functions of myCAFs that crucially affected the carcinogenesis, prognosis, and therapy responsiveness of BLCA. Further studies on myCAFs may provide potential diagnostic biomarkers and therapeutic targets for BLCA.

## Materials and Methods

### Raw Data Acquisition

The gene transcriptome data of 408 patients with BLCA were downloaded from the TCGA portal (https://portal.gdc.cancer.gov/), 406 patients with complete clinical information were further selected. The average gene expression of the samples from the same patient was calculated using the “limma” package of R software version 4.0.3. Gene expression was transformed into TPM for further analysis. Meanwhile, GEO cohorts (GSE13507 and GSE32894) were obtained from the Gene Expression Omnibus (https://www.ncbi.nlm.nih.gov/geo/). Gene expression of the TCGA and GEO cohorts was transformed by log2 (expression+1) before normalization by the “Combat” algorithm of the “SVA” package. The integrated cohort (TCGA, GSE13507, and GSE32894) was used for subsequent analysis. The IMvigor210 cohort was obtained from the R package “IMvigor210CoreBiologies” for external validation (Mariathasan et al., 2018). (http://researchpub.gene.com/IMvigor210CoreBiologies/IMvigor210CoreBiologies.tar.gz). The information of the cohorts used in this manuscript was provided in Table 1.

**TABLE 1:** Information for the BLCA cohorts used in the present study.

Cohort	GSE13507	GSE32894	TCGA-BLCA	IMvigor210
Type (Number)	NMIBC(103), MIBC(62)	NMIBC(215), MIBC(93)	NMIBC(5), MIBC(401)	
Gender	Female:30, Male:135	Female:80, Male:228	Female:107, Male:299	Female:65, Male:233
Survival outcome	OS	DFS	OS	

### Weighted Gene Co-Expression Network Analysis (WGCNA) and Differentially Expressed Genes (DEGs) Analysis

The “WGCNA” R package was used to identify the genes correlated with normal and tumor tissues for co-expression network analysis. A heatmap displays the values of the correlation between each module and the normal and tumor tissues. The genes with the highest correlations in the modules were selected for subsequent analysis. DEGs were calculated using the Limma package of R software between different groups and were defined as genes with adjusted *p*-value < 0.05 and |Log2 (Fold Change)| >1.

### Survival Analysis and Independent Prognostic Factor Screening

Kaplan–Meier (KM) survival analysis with the log-rank test was used to compare the survival differences in the present study. Figures were plotted using the R packages “survival” and “survminer.” The Univariate and multivariate Cox regression analyses were performed to screen the independent risk factors for patients’ overall survival (OS) and disease-free survival (DFS). Due to the different survival information in the integrated BLCA cohort, the survival analysis for OS in this study collected patients from the TCGA and GSE13507 cohorts. The analysis for DFS included patients from the GSE32894 cohort.

### Consensus Cluster

Consistency analysis of all the samples was conducted using the ‘ConsensusClusterPlus’ package of R software version 4.0.3. The maximum number of clusters was 9, and 80% of the total sample was drawn 50 times, clusterAlg = “km,” distance = “euclidean.” In this study, BLCA patients were clustered into three distinct subgroups based on the gene expression levels of myCAF marker genes, including ACTA2, TAGLN, MYL9, TPM1, and TPM2.

### Gene Ontology and Kyoto Encyclopedia of Genes and Genomes Pathway Enrichment Analysis

Gene Ontology (GO), which included molecular function (MF), biological pathways (BP), and cellular components (CC), was used for functional annotation. Kyoto Encyclopedia of Genes and Genomes (KEGG) pathway analysis was used to obtain an analytical study based on DEGs. The “ClusterProfiler” package of R software was used to annotate GO functions and enrich the KEGG pathways.

### Calculation of the Cancer Stemness Index

The OCLR algorithm was constructed by Malta et al. to calculate cancer stemness based on mRNA expression and DNA methylation levels (Malta et al., 2018). In this manuscript, we used EREG-mRNAsi and EREG-DNAsi to represent the stemness of each sample. We further corrected the stem cell index by adjusting to the tumor purity and obtained the corrected stem cell index (mRNAsi or DNAsi/TumorPurity). The cancer stemness results of TCGA patients were obtained from the UCSC Xena database (https://xenabrowser.net/datapages/).

### Gene Set Variation Analysis and Single-Sample Gene Set Enrichment Analysis (ssGSEA)

Gene set variation analysis (GSVA) is a pathway-based analysis method that provides each sample with an overall pathway or gene set activity score (Hänzelmann et al., 2013). The pathways of the hallmark gene sets were used for GSVA to identify their comprehensive activities. The ssGSEA algorithm is a rank-based method defining a score representing the degree of absolute enrichment of a particular gene set in each sample. We constructed the myCAFs score based on the combined expression of myCAFs marker genes, including ACTA2, TAGLN, MYL9, TPM1, and TPM2. The GSVA and ssGSEA processes were conducted by the R Bioconductor package Gene Set Variation Analysis version 3.5. The high and low myCAFs groups were classified based on the medium value of the myCAFs score.

### Bladder Cancer Molecular Subtyping

The molecular subtype of BLCA was obtained from previously published articles, which classified BLCA into five subtypes (Robertson et al., 2017) according to molecular expression, mutation, and immune infiltration.

### Estimation of Tumor-Infiltrating Immune Cells and Biological Functions

The abundance of the TME components was estimated by the ‘immunedeconv’ (Sturm et al., 2019) R package. The results of four different algorithms, including TIMER (Li et al., 2016), CIBERSORT (Newman et al., 2015), xCELL (Aran et al., 2017), and MCP-COUNTER (Becht et al., 2016), are displayed. Subsequently, scores of biological functions, including lipid metabolism (Wu et al., 2019), energy metabolism (Zhou et al., 2018), DNA repair (Jinjia et al., 2019), senescence-associated secreting phenotype (SASP) (Birch and Gil, 2020), and ageing (Cardoso et al., 2018), were acquired through the ssGSEA algorithm.

### Calculation of Tumor Immune Dysfunction and Exclusion and Prediction of ICB Treatment Reactiveness

The tumor dysfunction and exclusion scores of each patient were calculated using the TIDE algorithm. Potential ICB response was predicted based on the dysfunction and exclusion score. TIDE uses various gene expression markers to assess two distinct tumor immune escape mechanisms, including tumor-infiltrating cytotoxic T lymphocyte (CTL) dysfunction and exclusion by immunosuppressive factors. Higher TIDE scores indicated poorer efficacy of ICB therapy (Jiang et al., 2018).

### Prediction of the Chemotherapeutic Response

We predicted the chemotherapeutic response for each TCGA sample based on the largest publicly available pharmacogenomics database, the Genomics of Drug Sensitivity in Cancer (GDSC), (https://www.cancerrxgene.org). The prediction process was implemented by the R package “pRRophetic,” where the half-maximal inhibitory concentration (IC50) of the samples was estimated by ridge regression (Geeleher et al., 2014; Lu et al., 2019).

### Gene Mutation Analysis

Somatic mutation information was downloaded from the TCGA database and subsequently visualized using the R package “maftools.” The waterfall plot showed mutation data of each gene. The specific mutation types were annotated with different colors at the bottom left of the waterfall plot. The tumor mutation burden (TMB) was estimated as (total mutation/total covered bases) × 10^6.

### Immunohistochemical Analysis and Scoring

Forty postoperative BLCA sections from 2016 to 2021 were recruited for IHC analysis with the approval of the institutional ethics committee. The patients’ clinical information is listed in the following table (Table 2). The BenchMark GX automatic immunohistochemical staining system (Roche, Switzerland) with the Opti View DAB Detection Kit (Ventana, USA) was used to detect ACTA2 (Abcam, catalog number: ab7817, 1:250) expression in this study. The primary antibodies were visualized using a horseradish peroxidase-labeled secondary antibody. Hematoxylin was applied for counterstaining, whereas Bluing Reagent was applied for post counterstaining. The mean integrated optical density (IOD) values of positive protein expression was calculated by Image-Pro Plus 6.0.

**TABLE 2:** Clinical information for BLCA patients with IHC analysis.

Characteristics	Age		Gender		T Stage		N stage		M Stage		Grade	
	≤65	>65	Male	Female	Ta-T1	T2-T4	N−	N+	M−	M+	High	Low
Number	19	21	30	10	26	14	38	2	37	3	32	8

### Statistical Analysis

Principal component analysis (PCA) confirmed the distinct distribution of the clusters gained from the consensus analysis. The Wilcoxon test was used to examine the differences between variables of the two groups. Furthermore, the Kruskal–Wallis test (non-normal distribution) or one-way ANOVA (normal distribution) was used to analyze statistically significant differences for the variables of more than two groups. The Spearman correlation test examined the relationship between two different elements. The Receiver operating characteristic (ROC) curves were used to determine the predictive accuracy of myCAFs marker genes for bladder carcinogenesis. A two-sided *p* value < 0.05 was considered statistically significant. All statistical analyses were performed using R language v4.0.3.

## Results

### Five myCAFs Marker Genes Were Identified to Be Associated With the Pathogenesis and Prognosis of BLCA

**FIGURE 1:**
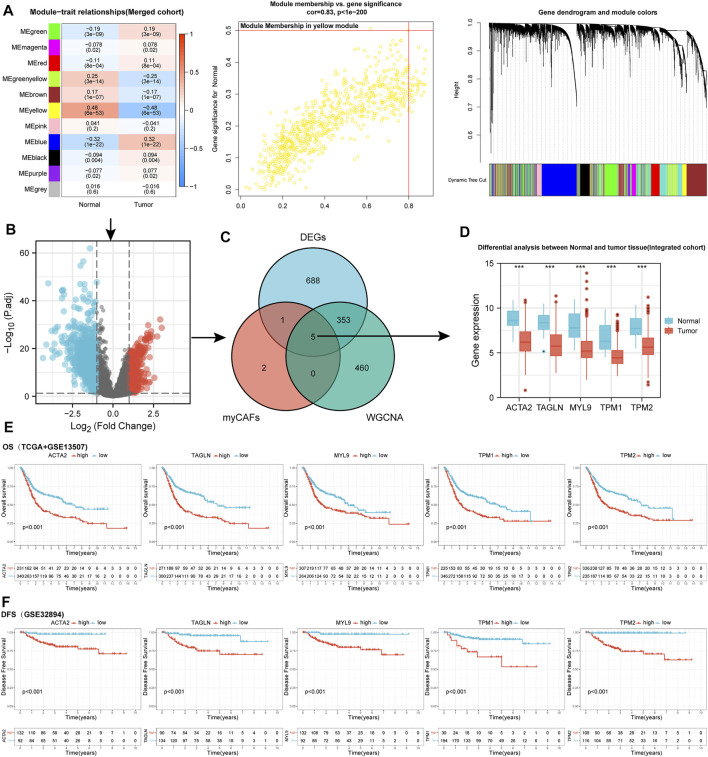
WGCNA and DEGs analysis identified five marker genes of myCAFs that showed bimodal functions on bladder cancer carcinogenesis and prognosis. **(A)** WGCNA indicated the yellow module was the most correlated module with bladder carcinogenesis, with a correlation efficiency (Gene significance to module membership) of 0.83. **(B)** Volcano plot displayed the differentially expressed genes between normal and tumor samples with adjusted *p*-value<0.05 and |Log2(Fold Change)|>1. **(C,D)** The intersection of WGCNA and DEG analysis in the integrated cohort identified the expression of five myCAFs related genes, including ACTA2, TAGLN, MYL9, TPM1, and TPM2, were significantly down-regulated in tumor tissue (*p* < 0.001). **(E,F)** KM survival analysis revealed that patients with high expression of ACTA2, TAGLN, MYL9, TPM1, and TPM2 owned shortened OS (*p* < 0.001) (panel **E**) and DFS (*p* < 0.001) (panel **F**). ***p < 0.001.

We applied WGCNA and DEGs analysis between normal and BLCA samples to screen for genes potentially associated with bladder carcinogenesis. The yellow module of WGCNA was identified as the most related module with tumorigenesis (Figure 1A). A total of 325 up-regulated and 722 down-regulated DEGs were identified by differential analysis (Figure 1B). After intersecting genes in the yellow module of WGCNA with DEGs, we identified five myCAFs marker genes defined by previous single-cell sequencing (Elyada et al., 2019) that were significantly down-regulated in tumor tissues, including ACTA2, MYL9, TAGLN, TPM1, and TPM2 (Figures 1C,D). To clarify the impact of these genes on the prognosis of BLCA, we applied the KM survival analysis, with the results showing that high expression of these genes impaired the OS (Figure 1E) and DFS (Figure 1F) of BLCA patients. These results indicated the dual roles of myCAFs marker genes in tumorigenesis and BLCA prognosis.

### Consistent Clustering Arranged BLCA Patients Into Three Distinct Subgroups Based on the Expression of the Five myCAF Marker Genes

**FIGURE 2:**
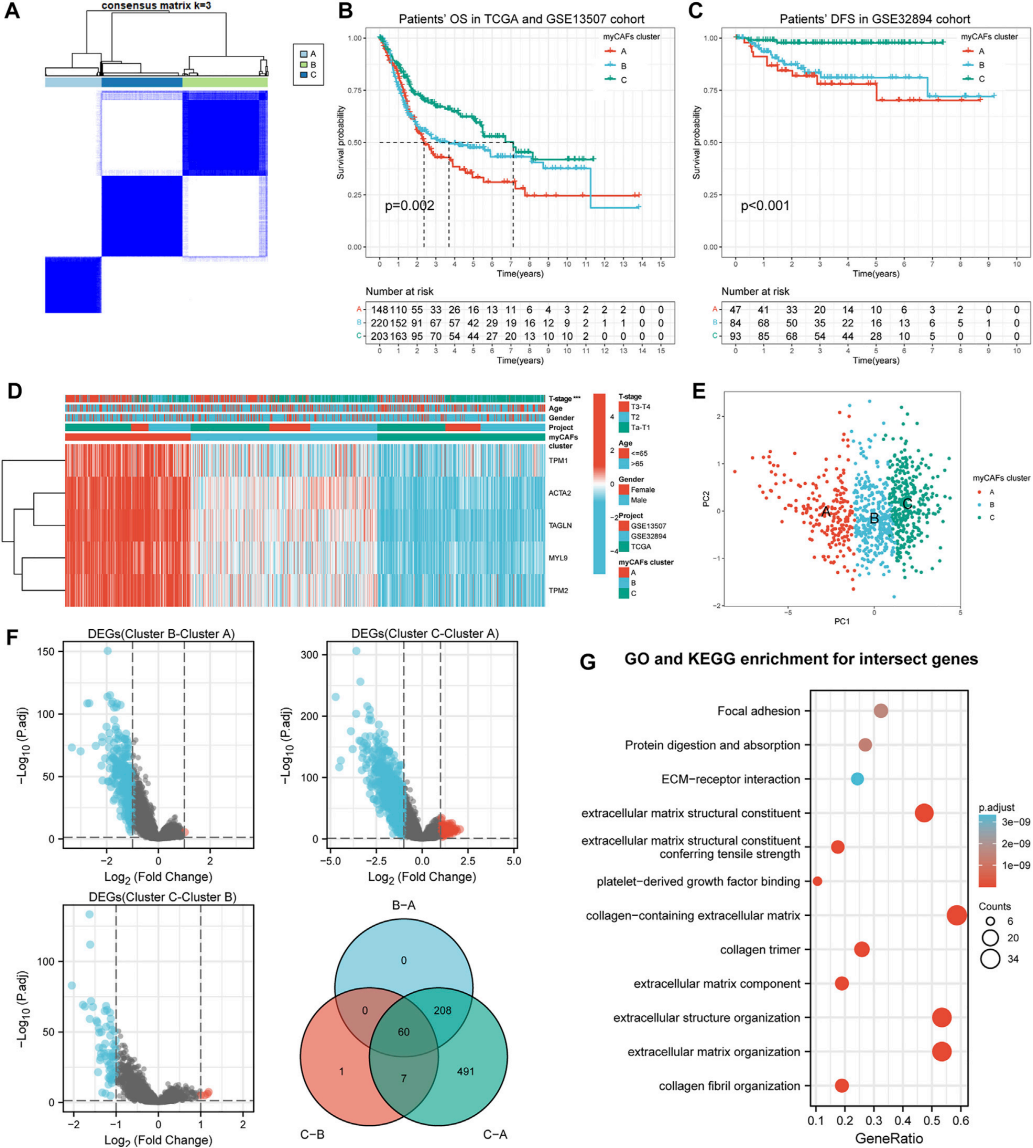
Consensus clusters identified three subgroups of BLCA patients showing significant differences in clinical pathological and tumor microenvironment features. **(A)** BLCA patients in the integrated cohort were classified into three distinct clusters based on the myCAFs related gene expressions. **(B)** KM survival analysis indicated a significant OS difference between the three clusters (*p* = 0.002), with patients of cluster A showing the lowest medium survival interval. **(C)** Patients’ DFS was significantly lower in cluster A and B than that in cluster C (*p* < 0.001). **(D)** The heatmap demonstrated the association of BLCA subgroups with T-stage, age, and gender, suggesting that subgroups with high levels of myCAFs have a higher proportion of more advanced BLCA patients (*p* < 0.001). **(E)** Principal component analysis confirmed the scattered distribution of CAFs related gene expressions between the three clusters. **(F,G)** The 60 common differentially expressed genes between the three clusters were significantly enriched in extracellular matrix remodeling related processes, including extracellular structure organization, ECM-receptor interaction, and Focal adhesion.

We subsequently integrated the gene expression data of BLCA patients from the TCGA, GSE13507, and GSE32894 cohorts. Consistent clustering classified the BLCA patients from the integrated cohort into three subgroups based on these five myCAFs marker genes (Figure 2A). Patients’ OS (*p* = 0.002) and DFS (*p* < 0.001) significantly differed between the three clusters (Figures 2B,C), suggesting that the different abundance of myCAFs may impact the prognosis of BLCA patients. By analyzing the clinical characteristics of patients in different subgroups, we found that the proportion of more advanced BLCA was higher in the cluster with higher myCAFs content (*p* < 0.001), suggesting a potential association of myCAFs abundance with the T-stage of BLCA (Figure 2D; Table 3). Subsequently, we confirmed the different expression levels of myCAFs-related genes in these three groups using PCA (Figure 2E). By screening the DEGs among the three subgroups, we further identified a total of 60 common DEGs (Figure 2F) that were significantly involved in extracellular matrix remodeling, further confirming that the abundance of myCAFs may indeed confer different clinical and TME features to these three subgroups of BLCA patients (Figure 2G).

**TABLE 3:** Different invasive features among distinct BLCA subgroups.

Cluster	T3-T4	T2	Ta-T1
Cluster A	107 (48.4%)	70 (31.7%)	44 (19.9%)
Cluster B	121 (36.8%)	83 (24.2%)	125 (38.0%)
Cluster C	62 (20.9%)	82 (27.7%)	152 (51.4%)

Chi-square test *p*-value < 0.001.

### myCAFs Abundance Was an Independent Risk Factor for Patients' OS and DFS

**FIGURE 3:**
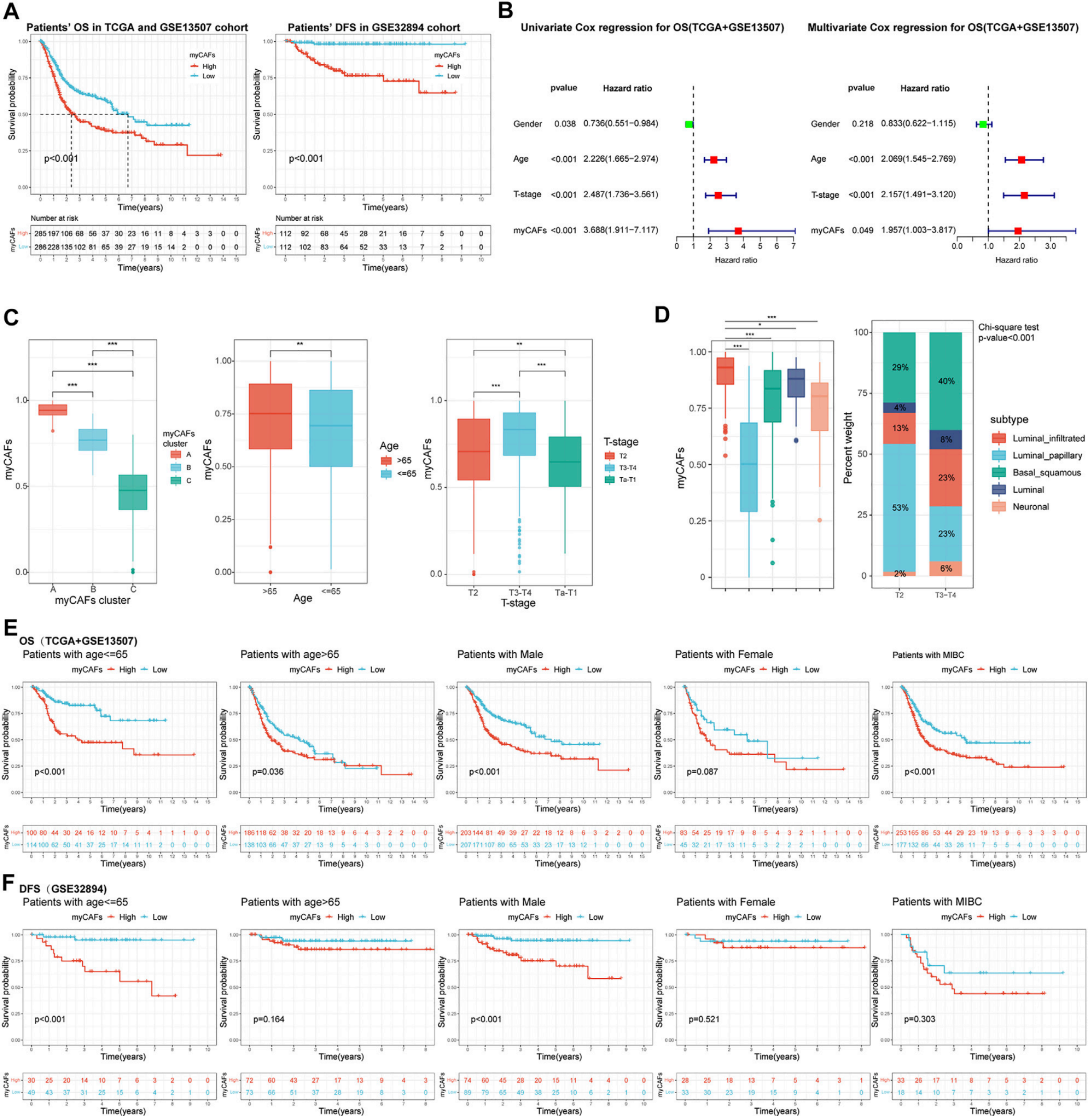
The myCAFs score was significantly correlated with patients’ prognosis and clinical-pathological features. **(A)** Patients with high myCAFs scores showed significant impaired OS (*p* < 0.001) and DFS (*p* < 0.001). **(B)** The myCAFs score acted as an independent risk factor for patients’ OS revealed by multivariate Cox regression analysis (*p* = 0.049). **(C)** The myCAFs score was significantly correlated with patients’ myCAFs cluster, age, and T-stage. **(D)** Significant differences in myCAFs scores were found among molecular subtypes of BLCA, with a higher proportion of myCAFs-rich molecular subtypes in more advanced BLCA (*p* < 0.001). **(E)** Subgroup survival analysis in TCGA and GSE13507 cohorts showed that patients with high myCAFs scores tended to own impaired OS, especially patients with male (*p* < 0.001) and age less than 65 (*p* < 0.001). **(F)** Subgroup survival analysis on patients’ DFS in the GSE32894 cohort also confirmed high myCAFs score shortened DFS in patients with male (*p* < 0.001) and age less than 65 (*p* < 0.001). ***p < 0.001, **p < 0.01, *p < 0.05.

To better quantify the abundance of myCAFs, we constructed a myCAF score by the ssGSEA algorithm based on the combined expression levels of the five myCAF marker genes. High myCAFs score significantly shortened patients’ OS (high vs. low, *p* < 0.001) and DFS (high vs. low, *p* < 0.001) (Figure 3A) and acted as an independent risk factor for OS (multivariate Cox regression, *p* = 0.049) (Figure 3B) and DFS (multivariate Cox regression, *p* = 0.031) (Table 4). The correlation analysis between myCAFs scores and clinical characteristics of BLCA patients revealed significant differences in myCAFs scores between subgroups of myCAFs, between BLCA patients with different ages and T-stages (Figure 3C). These results suggested that advanced BLCA tended to own higher myCAFs abundance. Meantimes, we found that the subtypes with higher myCAFs scores took a higher proportion in T3-T4 patients than in T2 patients, laterally suggesting the potential correlation of myCAFs abundance with the T-stage of BLCA patients (Figure 3D). We also observed that the patient’s age crucially altered the myCAFs score, indicating that senescence impacts the abundance of myCAFs. To further discuss the effect of myCAFs on survival in different subgroups of BLCA patients, we conducted subgroup survival analysis and found that the myCAFs level had a significant impact on the OS and DFS of BLCA patients in several subgroups, especially in young (age <65) and male patients (Figures 3E,F).

**TABLE 4:** Univariate and multivariate Cox regression of myCAFs for patient DFS.

Characteristics	HR	HR.95 L	HR.95H	*p*-value
Univariate Cox regression				
Gender	1.538	0.577	4.100	0.389
Age	0.551	0.251	1.210	0.137
T-stage	45.087	10.617	191.473	<0.001
myCAFs	144.304	10.964	1899.241	<0.001
Multivariate Cox regression				
Gender	2.232	0.796	6.255	0.127
Age	0.636	0.276	1.464	0.287
T-stage	30.523	6.976	133.555	<0.001
myCAFs	20.181	1.320	308.566	0.031

### myCAFs regulated in T Cell Infiltration and Immune Response, Further Influencing the Immunotherapy Responsiveness of BLCA Patients

**FIGURE 4:**
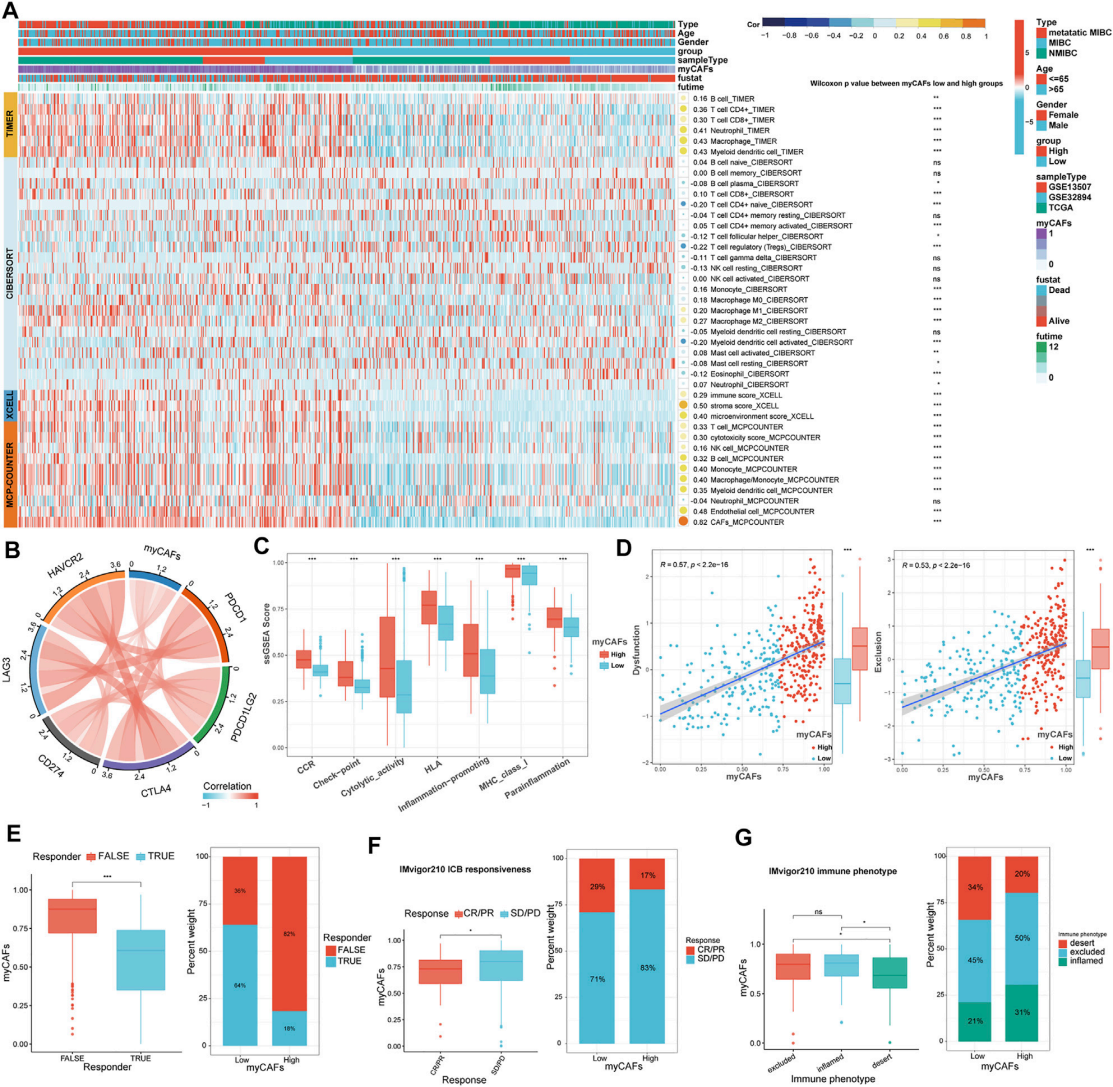
Relationship between myCAFs score and the immune landscape of BLCA patients. **(A)** The TIMER, CIBERSORT, xCELL, and MCP-COUNTER algorithms showed significantly higher CD8^+^ T cells (*p* < 0.001) and M2 macrophages (*p* < 0.001) in the high myCAFs group. **(B)** The myCAFs score was significantly positively correlated with the expression levels of multiple immune checkpoint-related genes, including CD274 (R = 0.21, *p* < 0.001), PDCD1 (R = 0.28, *p* < 0.001), CTLA4 (R = 0.40, *p* < 0.001), PDCD1LG2 (R = 0.45, *p* < 0.001), HAVCR2 (R = 0.47, *p* < 0.001), and LAG3 (R = 0.33, *p* < 0.001). **(C)** Immune-related functions, including CCR (*p* < 0.001), checkpoint (*p* < 0.001), cytotoxic activity (*p* < 0.001), HLA (*p* < 0.001), inflammation-promoting (*p* < 0.001), MHC_class_I (*p* < 0.001), and para inflammation (*p* < 0.001) were significantly higher in patients with high myCAFs scores. **(D)** The myCAFs score was positively correlated with T cell dysfunction (R = 0.57, *p* < 0.001) and exclusion score (R = 0.53, *p* < 0.001) gained from the TIDE algorithm. **(E)** TIDE algorithm predicts that patients with high myCAFs scores are more likely to be unresponsive to ICB treatment (*p* < 0.001). **(F)** The results of the IMvigor210 immunotherapy cohort confirmed the lower responsive rate of high myCAFs score patients. **(G)** myCAFs scores were higher in excluded (*p* < 0.05) and inflamed (*p* < 0.05) phenotype than that in desert phenotype. ***p < 0.001, **p < 0.01, *p < 0.05, ns: not significant.

With the TIMER, CIBERSORT, and MCP-COUNTER algorithms, we found that tumors with high myCAFs scores had higher CD8^+^ T cell infiltration and possessed elevated levels of immunosuppressive cells such as M2 macrophages (Figure 4A). myCAFs scores were also correlated with tumor microenvironment scores provided by xCEll, especially stromal scores. These results suggest that the abundance of myCAFs in stromal components may increase the infiltration of CD8^+^ T cells and affect the M2 polarization of macrophages. Further analysis of the immune processes associated with myCAFs revealed that myCAFs scores were significantly positively correlated with the levels of various immune checkpoint molecules (Figure 4B). Tumors with high myCAFs scores had higher CCR, checkpoint, cytotoxicity, HLA, MHC, and proinflammatory activities (Figure 4C). The TIDE algorithm further revealed that myCAFs scores significantly correlated with T cell exclusion and dysfunction (Figure 4D). These results indicated that myCAFs could induce and sequester CD8^+^ T cells in the tumor microenvironment, hinder their infiltration into the tumor tissue, and ultimately lead to T cell dysfunction, resulting in immune evasion. Subsequent predictions of response to immune checkpoint inhibitor therapy by the TIDE algorithm confirmed these inferences, showing that patients with high myCAFs scores exhibited lower responsiveness to immune checkpoint therapy (Figure 4E). Immune checkpoint responsiveness results from the IMvigor210 cohort further confirmed the predictions of the TIDE algorithm (Figure 4F). We also validated the characteristics of myCAFs scores in patients with different T cell infiltration features in the IMvigor210 cohort. Specifically, myCAFs scores were higher in the inflamed and excluded phenotypes than in the desert phenotype (Figure 4G).

### myCAFs Were Correlated With Tumor Metabolic Features, Senescence-Associated Secreting Phenotype, and Cancer Stemness, Influencing the Responsiveness of BLCA Patients to Chemotherapy

**FIGURE 5:**
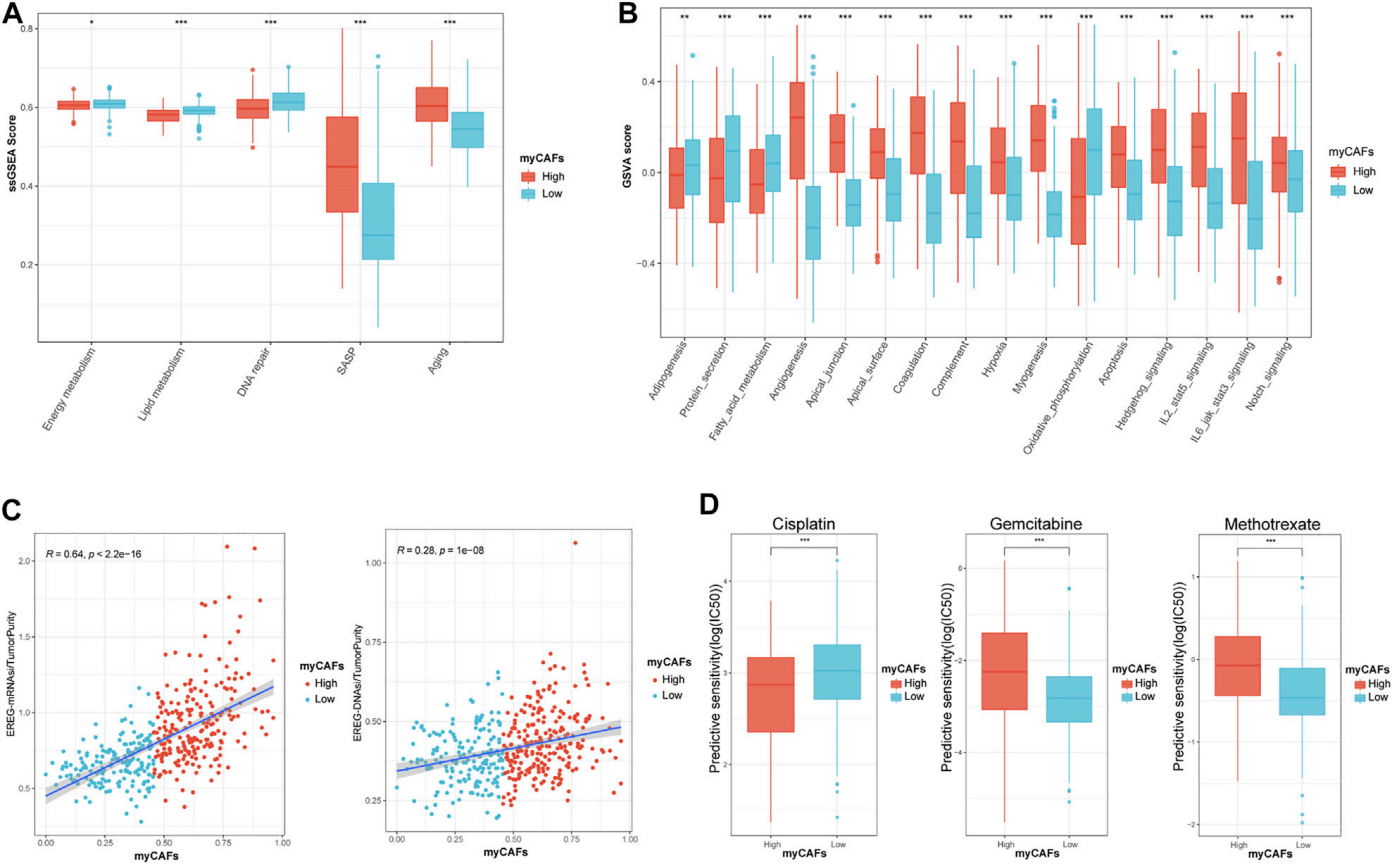
myCAFs score was correlated with tumor metabolic activities, senescence, tumor stemness, and chemotherapy responsiveness in BLCA patients. **(A,B)** myCAFs are closely associated with energy (*p* < 0.05), lipid (*p* < 0.001), fatty acid (*p* < 0.001) metabolic activities, senescence-related secretory phenotypes (*p* < 0.001), tumor microenvironment remodeling-related processes (*p* < 0.001), and cancer stemness-related pathways (*p* < 0.001). **(C)** The myCAFs score was positively correlated with EREG mRNAsi (R = 0.64, *p* < 0.001) and EREG DNAsi (R = 0.28, *p* < 0.001), indicating that the myCAFs score was positively correlated with cancer stemness. **(D)** The responsiveness of chemotherapy predicted by the “pRRophetic” package highlighted patients with high myCAFs scores tend to benefit from cisplatin chemotherapy (*p* < 0.001) but are resistant to gemcitabine (*p* < 0.001) and methotrexate (*p* < 0.001). ***p < 0.001, **p < 0.01, *p < 0.05.

We further analyzed the metabolic characteristics associated with myCAFs. We found that energy, lipid metabolism, fatty acid metabolism, and adipogenesis activities were significantly reduced in tumors with high myCAFs. In contrast, SASP and senescence-related gene expression were significantly more active in tumors with high myCAFs. After GSVA, we found that tumors with high myCAFs exhibited stronger angiogenesis and myogenesis, more elevated hypoxia, and lower oxidative phosphorylation levels. These results fully suggested that remodeling of the tumor microenvironment by myCAFs could occur through multiple pathways and finally result in a hypoxia- and nutrition-deprived tumor microenvironment. In addition, myCAFs could also affect several signaling pathways related to tumor cell stemness, such as the STAT3 (Wang et al., 2018), KRAS (Yoon et al., 2019), and Notch (Yang et al., 2020) signaling pathways, suggesting that the abundance of myCAFs may affect tumor stemness (Figures 5A,B). We then performed correlation analysis between myCAFs and tumor stemness by two different scoring algorithms, the mRNA stemness index (EREG-mRNAsi) and the DNA stemness index (EREG-DNAsi). We corrected the above index based on tumor purity, considering the influence of stromal components (Stemcell index/TumorPurity). The results showed that the myCAFs score had a significant positive correlation with the tumor stemness index, suggesting that tumor stemness was more robust in patients with higher myCAFs scores (Figure 5C). Among the sensitivity of chemotherapeutic drugs obtained by the “pRRophetic” package, we found that patients with higher myCAFs scores tended to respond to cisplatin-based chemotherapy. In contrast, myCAFs abundance significantly impacted patient sensitivity to two commonly used chemotherapeutic drugs for BLCA, gemcitabine and methotrexate (Figure 5D).

### myCAFs Were Associated With Tumor Mutation Burden and Oncogenic Mutations, Especially FGFR3 and the RTK-RAS Signaling Pathway

**FIGURE 6:**
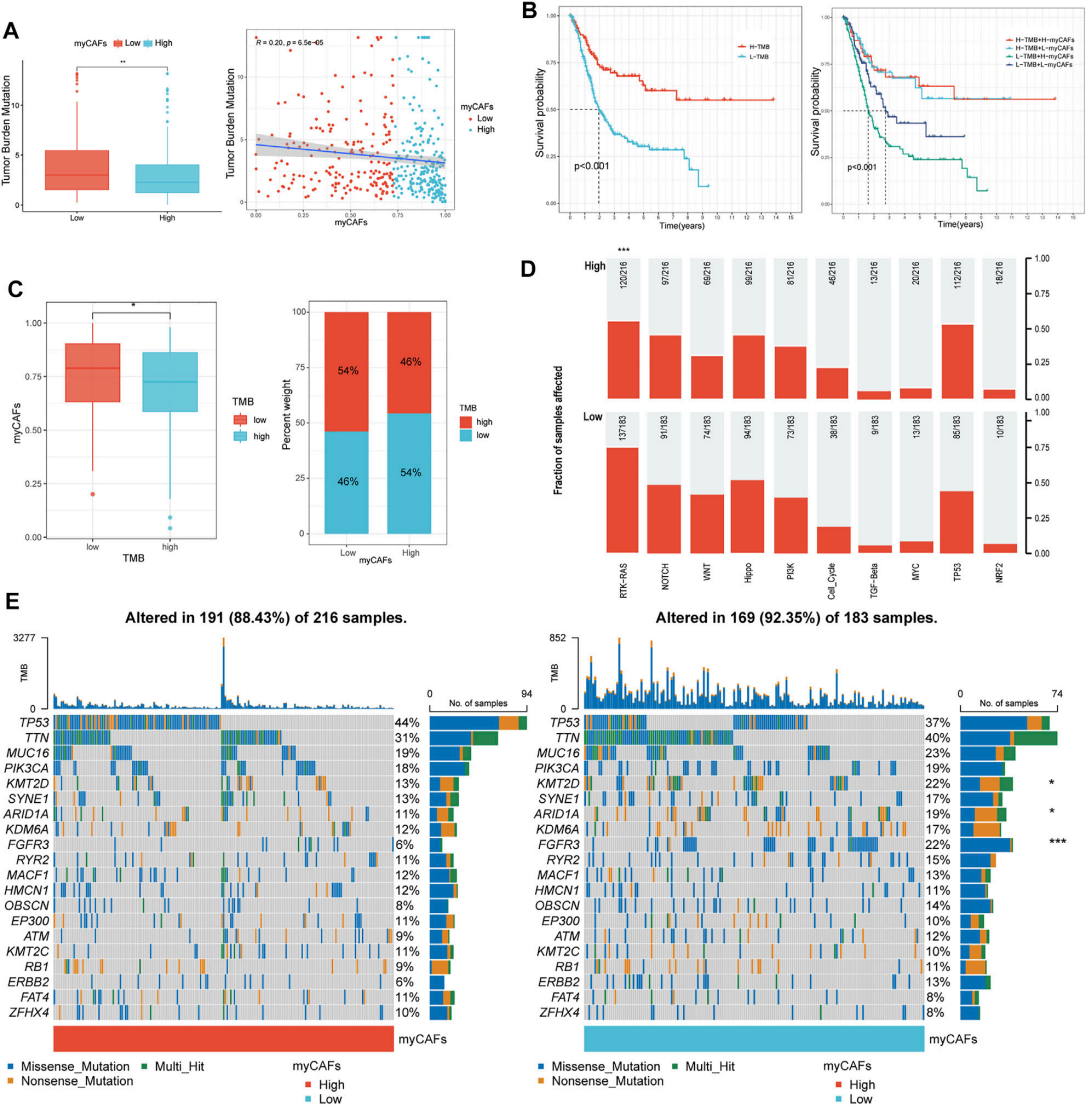
myCAFs score was related to the TMB and mutation frequency of FGFR3 and the RTK-RAS pathway. **(A–C)** myCAFs were negatively correlated with TMB (R = 0.20, *p* < 0.001), and the combination of myCAFs and TMB better differentiated patients’ OS (*p* < 0.001). The IMvigor210 cohort validated the negative correlation between myCAFs and TMB in the TCGA cohort (*p* < 0.05). **(D)** the mutation frequency of the RTK-RAS signaling pathway was higher in the low myCAFs score group (*p* < 0.001). **(E)** the waterfall plot showed lower FGFR3 (*p* < 0.001), ARID1A (*p* < 0.05), KMT2D (*p* < 0.05) mutation frequency in high myCAFs score patients. ***p < 0.001, **p < 0.01, *p < 0.05.

After analyzing the gene mutation profile of the TCGA BLCA cohort by the “maftools” package, we combined the gene mutation information with myCAFs scores and found that myCAFs scores conformed to show a significant negative correlation with tumor mutation burden (TMB) (Figure 6A). In addition, myCAFs score combined with TMB had a more substantial effect on patients' OS (Figure 6B), and the mutation information of the IMvigor210 cohort further confirmed the negative correlation between myCAFs abundance and TMB (Figure 6C). In addition, the abundance of myCAFs had a significant effect on the RTK-RAS signaling pathway (Figure 6D) and FGFR3 mutation frequency (Figure 6E), showing that tumors with high myCAFs scores often possessed lower RTK-RAS signaling pathways and FGFR3 mutation frequencies.

### IHC Analysis Validated the Dynamics of myCAFs During Tumor Development

**FIGURE 7:**
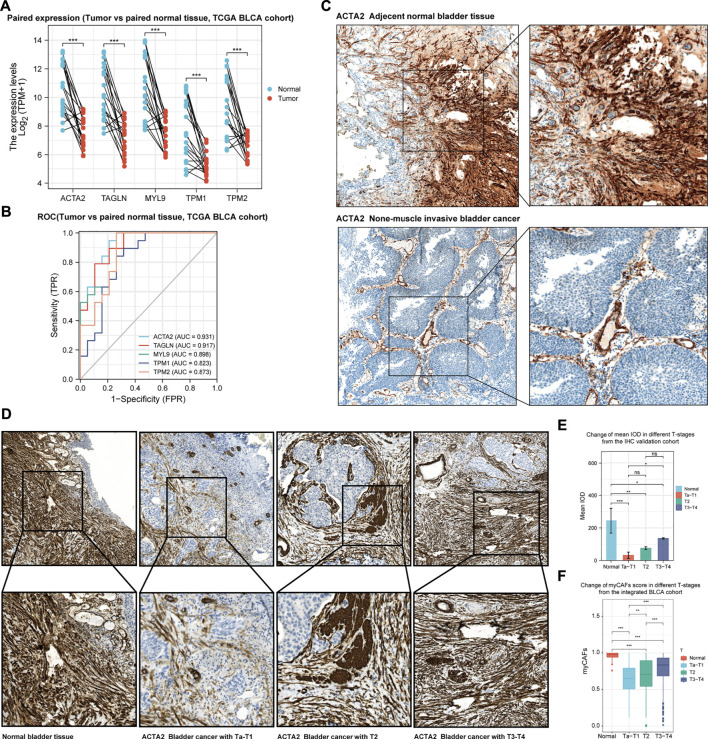
The development of bladder cancer is accompanied by dynamic changes in the abundance of myCAFs. **(A)** myCAFs related gene expressions were significantly reduced in BLCA samples compared with adjacent normal tissue (*p* < 0.001) in the TCGA BLCA cohort. **(B)** The ROC curve demonstrated that decreased expression of the five myCAFs marker genes was highly accurate in predicting bladder carcinogenesis. **(C)** IHC analysis confirmed the reduction of myCAFs in BLCA tissue compared with paired normal tissue. **(D,E)**. IHC with IOD analysis suggested a significant decrease of myCAFs abundance in tumor samples (normal to Ta-T1, *p* < 0.001; normal to T2, *p* < 0.01; normal to T3-T4, *p* < 0.05), while high myCAFs abundance was observed in advanced BLCA sections (T3-T4 to Ta-T1, *p* < 0.05). **(F)** ssGSEA generated myCAFs score in the integrated BLCA cohort confirmed the dynamic change of myCAFs abundance in the development of BLCA. ***p < 0.001, **p < 0.01, *p < 0.05, ns: not significant.

Combined with the above bioinformatics analysis, we found a dynamic change in myCAFs abundance during the development of BLCA. To validate this phenomenon, we further compared the expression levels of the myCAFs marker genes in paired BLCA and normal paraneoplastic samples in the TCGA cohort (Figure 7A). The results confirmed the reduced expression of the myCAFs marker genes in tumor tissues. ROC analysis indicated highly diagnostic accuracy of the myCAFs marker genes in distinguishing tumors from normal bladder tissues, especially ACTA2 (Figure 7B). Through IHC assay, we verified the reduced ACTA2 expression levels in tumor tissues compared with the adjacent normal mucosa, indicating a decreased myCAFs abundance in BLCA tissue (Figure 7C). Further analysis of ACTA2 in BLCA sections with different stages revealed that ACTA2 expression levels were significantly elevated in T2 (MIBC without external bladder invasion) and T3-T4 BLCA (MIBC with external bladder invasion) (Figure 7D). Thus, we summarize the characteristics of changes in ACTA2 expression during BLCA carcinogenesis and progression in our recruited postoperative sections (Figure 7E), showing similar features to the dynamic changes in the fraction of myCAFs in our bioinformatics analysis cohorts (TCGA, GSE13507, and GSE32894) (Figure 7F).

## Discussion

BLCA is the fourth most common cancer among the male population (Kaneko and Li, 2018). The incidence rate of BLCA is higher in men, three to five times higher than that in women. It has a worldwide incidence and mortality of 330,000 and 123,000, respectively (Davis et al., 2018). Therefore, BLCA is a significant burden on global public health. Most BLCAs are urothelial carcinomas and are classified as either NMIBC or MIBC because of the distinct implications on patient management. Radical cystectomy is still the mainstay treatment for MIBC. Treatments for high-grade muscle invasive and metastatic BLCA have not advanced beyond gemcitabine and cisplatin combined chemotherapy. Recently, ICB therapy has opened the possibility of immunotherapy for BLCA, especially for muscle-invasive and metastatic BLCA, when chemotherapy fails.

While cancer originates from the accumulation of mutations within cancer cells, cancer progression and therapy responsiveness are strongly modulated by the surrounding stromal cells in the tumor microenvironment (Sahai et al., 2020). The last decades have witnessed a significant research trend towards CAFs. It is now believed that CAFs regulate cancer proliferation and metastasis through growth factor production, synthesis, and remodeling of the extracellular matrix (ECM). Recently, there has also been a growing understanding of the ability of CAFs to regulate the immune system. Targeting CAFs, by altering their number, subtype, or function is being explored to improve cancer therapy (Sahai et al., 2020). With the advancement of research techniques in recent years, many controversies have emerged in the study of CAFs, including the different effects of CAFs on tumors.

While most previous reports showed a significant tumor-promoting effect of CAFs, studies of CAFs have also identified the antitumor roles of CAFs (Özdemir et al., 2014). Evidence proposed that tumor stroma played a bimodal role in cancer development, impeding neoplastic growth in normal tissue while encouraging migration and tumor growth during tumor progression. The heterogeneity of CAFs allows them to comprise multiple subgroups, including tumor-promoting and tumor-suppressing CAFs (Schauer et al., 2011). As the fibroblasts are very heterogeneous and highly plastic, temporal changes of the tumor microenvironment could dramatically affect the dynamics of fibroblasts during cancer development. It has been demonstrated that there is a process of interconversion between different subgroups of CAFs and that the conversion of cancer-inhibiting to cancer-promoting CAFs may accompany the development of BLCA (Li B. et al., 2021). A recent study also demonstrated that CAFs in lung metastases are transcriptionally dynamic and plastic, revealing stage-specific gene signatures of CAFs that imply functional tasks to remodel the tumor microenvironment, including extracellular matrix remodeling, stress response, and shaping the inflammatory microenvironment (Shani et al., 2021). By investigating the effects of myofibroblasts in early lesions in breast cancer development and progression, Betul G et al. revealed the phenotypic and functional characteristics of CAFs in preneoplastic lesions, further underlining the importance of temporal changes in CAFs during cancer progression (Gok Yavuz et al., 2018). Freja A et al. demonstrated that multiple subpopulations of CAFs co-exist in murine breast cancer and that the abundance and dynamics for each marker differ depending on tumor type and time (Venning et al., 2021). In the present article, we also found evidence with the bioinformatics analysis supporting that myCAFs played dual functions within the carcinogenesis and progression of BLCA, further emphasizing the significance of the temporal change in tumor microenvironment on both the tumor and stromal cells. Meanwhile, Our study also found that the pathogenesis of epithelial tumors like BLCA was accompanied by a significant decrease in fibroblast content, resulting in significantly lower expression levels of marker genes for myCAFs such as ACTA2. However, it does not mean that activated fibroblasts are more deficient in BLCA than normal tissues. Previous studies have confirmed that the proportion of myCAFs in BLCA was still elevated compared to normal paraneoplastic tissues (Li B. et al., 2021). Therefore, it is critical to discuss the changes in fibroblasts during the development of BLCA in terms of their absolute content and the ratio of different CAFs subpopulations.

This present article identified five myCAFs marker genes that showed highly similar expression patterns and impact on BLCA patients’ prognosis, including ACTA2, TAGLN, MYL9, TPM1, and TPM2. Three different subgroups of BLCA patients based on the expression of these genes were revealed through systematic bioinformatics analysis. Patients with distinct myCAFs levels exhibited diverse tumor microenvironment features with altered infiltration of CD8 T cells and the polarization of M2 macrophages, further bringing differences in patients’ prognosis and treatment responses. With further verification of the TIDE algorithm and the IMvigor210 immunotherapy cohort, our results highlighted that the therapy targeting myCAFs might benefit patients’ therapy responsiveness and prognosis. Meantime, our study also indicated a crucial effect of senescence on the extracellular matrix and fibroblasts with elder patients possessing higher myCAFs abundances (Fane and Weeraratna, 2020), suggesting that aging-related factors also need to be adequately addressed in the study of CAFs.

In the present study, we also found that myCAFs were significantly correlated with the mutation frequency of FGFR3 in TCGA BLCA patients, showing that patients with high FGFR3 mutations possessed lower myCAFs abundance. We further revealed a negative correlation between myCAFs content and tumor mutation burden, suggesting that BLCA with high myCAFs content has a lower TMB. Since previous studies indicated that a low TMB is detrimental to the immune system’s recognition of tumor cells and affects the immunotherapeutic response, our results suggested that the effect of myCAFs on therapeutic responsiveness might also be related to tumor mutations (Sholl et al., 2020).

Predictive models based on bioinformatics analysis are widely available in the field of BLCA research, and many of them show high predictive accuracy (Abudurexiti et al., 2019; Li Z. et al., 2021; Du et al., 2021). However, there are still fewer diagnostic models for predicting the carcinogenesis of BLCA. In the present study, we constructed a myCAFs score that showed potential in distinguishing the pathogenesis of bladder cancer. Meanwhile, our myCAFs score has a significant predictive value for OS and DFS in BLCA. However, our prediction model did not show a considerable advantage in predictive accuracy on patients’ prognoses compared with other prognostic models. The main reason is that our myCAFs score was not constructed by the Cox regression model but by the ssGSEA algorithm. Precisely on this basis, our myCAFs can reflect the myCAFs content in each tumor sample more realistically than other prediction models, validated by the high correlation of our myCAFs score with the CAFs score calculated by the MCP-COUNTER algorithm (Figure 4A). At the same time, the ssGSEA algorithm integrates the combined expression of the five genes used for myCAFs score construction, leading to a high correlation of the myCAFs score with the expression levels of these five genes. In clinical applications, we can even detect one of these genes, especially ACTA2, to represent the myCAFs score, which is convenient for clinical applications and confirmed by our immunohistochemical experiments.

With our myCAFs score, we deeply explored the dynamic change of myCAFs during BLCA carcinogenesis and progression, suggesting that fibroblasts may play multiple roles in the process of tumor development in reaction to the time-dependent tumor microenvironment. However, our results were limited by the lack of deconvoluting algorithms that could precisely identify the accurate subgroups of CAFs in bulk sequencing. Further verification by scRNA-seq and experimental assays are required for discussing the temporal change of CAFs. Meantime, the prediction of therapy responsiveness in this manuscript mainly depended on bioinformatics algorithms and should be verified by further experimental and clinical research. In addition, we need a larger sample size of BLCA sections to validate the immunohistochemical results of this study.

## Conclusion

Our results revealed the dynamic changes in myCAFs abundance in the development of BLCA and highlighted the TME remodeling property of myCAFs, which further impacted BLCA patients’ therapy responsiveness and prognosis. The in-depth study of myofibroblasts can help explore the role of fibroblasts in the development of BLCA and provide possible diagnostic markers for predicting bladder carcinogenesis and potential therapeutic targets for BLCA treatment.

## Data Availability

The datasets presented in this study can be found in online repositories. The names of the repository/repositories and accession number(s) can be found in the article/supplementary material.
